# Immune-mediated cerebellar ataxias: from bench to bedside

**DOI:** 10.1186/s40673-017-0073-7

**Published:** 2017-09-21

**Authors:** Hiroshi Mitoma, Mario Manto, Christiane S. Hampe

**Affiliations:** 1 Tokyo Medical University, Medical Education Promotion Center, 6-7-1 Nishi-Shinjyuku, Shinjyuku-ku, Tokyo, 160-0023 Japan; 2 Unité d’Etude du Mouvement (UEM), FNRS, ULB-Erasme, 1070 Bruxelles, Belgium; 3 Service des Neurosciences, University of Mons, 7000 Mons, Belgium; 4 University of Washington, School of Medicine, Seattle, WA 98109 USA

**Keywords:** Cerebellar ataxias, Immune-mediated cerebellar ataxias, Diagnosis, therapy immunotherapy, Paraneoplastic cerebellar degeneration, Gluten ataxia, Anti-GAD65Ab-associated cerebellar ataxia

## Abstract

The cerebellum is a vulnerable target of autoimmunity in the CNS. The category of immune-mediated cerebellar ataxias (IMCAs) was recently established, and includes in particular paraneoplastic cerebellar degenerations (PCDs), gluten ataxia (GA) and anti-GAD65 antibody (Ab) associated-CA, all characterized by the presence of autoantibodies. The significance of onconeuronal autoantibodies remains uncertain in some cases. The pathogenic role of anti-GAD65Ab has been established both in vitro and in vivo, but a consensus has not been reached yet. Recent studies of anti-GAD65 Ab-associated CA have clarified that (1) autoantibodies are generally polyclonal and elicit pathogenic effects related to epitope specificity, and (2) the clinical course can be divided into two phases: a phase of functional disorder followed by cell death. These features provide the rationale for prompt diagnosis and therapeutic strategies. The concept “Time is brain” has been completely underestimated in the field of immune ataxias. We now put forward the concept “Time is cerebellum” to underline the importance of very early therapeutic strategies in order to prevent or stop the loss of neurons and synapses. The diagnosis of IMCAs should depend not only on Ab testing, but rather on a rapid and comprehensive assessment of the clinical/immune profile. Treatment should be applied during the period of preserved cerebellar reserve, and should encompass early removal of the conditions (such as remote primary tumors) or diseases that trigger the autoimmunity, followed by the combinations of various immunotherapies.

## Background

Autoimmune reactions against antigens in the central nervous system (CNS) often result in subtle and sometimes overt clinical symptoms. At an early stage, the identification of an immune process targeting the brain may be highly challenging. Multiple sclerosis (MS) is a representative autoimmune disease with a diffuse demyelination in the CNS. On the other hand, various diseases that target specific region(s) of the CNS have been identified [1, 2]. Interestingly, the limbic system and the cerebellum are two preferred targets of autoantibodies. During the last three decades, a clinical category of limbic encephalitis and immune-mediated cerebellar ataxias (IMCAs) has been established [1, 2].

The etiology of IMCAs varies considerably [2, 3]. Paraneoplastic cerebellar degeneration (PCDs) is a well-known autoimmune disorder of the cerebellum, characterized by the presence of specific autoantibodies against the associated neoplasm [4–9]. In addition, since the 1980s, a series of studies have described the clinical features of gluten ataxia (GA) [10] and anti-glutamate decarboxylase 65 antibody -associated CA (anti-GAD65Ab-associated CA) [11, 12]. Based on these studies, it is now clear that the true prevalence of IMCAs is higher than expected previously [13]. A large-scale study by Hadjivassiliou et al. reported that 30% of patients with CAs had IMCAs (25% had GA, 3% had PCDs, and 2% had anti-GAD65Ab-associated CA). On the other hand, 33% of the patients had gene deficiencies and 11% had multiple systemic atrophy (MSA) [13]. Interestingly, the association of autoantibodies to glutamate receptors has also been reported for CAs, as observed in limbic encephalitis [7–9].

To date, there is no consensus on the classification of autoimmune CAs. In our previous paper, we proposed a new classification based on (1) whether the cerebellum is the main target or not, and (2) whether the autoimmunity targeting the cerebellum is triggered by a specific condition or disease [2] (Table 1). While the pathophysiological mechanisms underlying the development of CAs still remain elusive in a proportion of cases, several studies have now clarified the challenges associated with the diagnosis, proposed various therapeutic strategies, and put forward guidelines based on the reported clinical cases (Table 2) [2, 3, 14]. The concept that “time is brain” was initially coined for stroke and emphasizes the rapid loss of nervous tissue occurring during a stroke [15]. In a stroke the neuronal loss is acute (from minutes to hours), while in the immune attack the loss is continuous (over days, weeks or months). Thus, there is growing concern that early therapeutic interventions should be considered to save cerebellar neurons also in case of immune attacks, since these events lead to irreversible neuronal loss. Quantitative estimates of the loss of neurons, synapses, and myelinated fibers in the cerebellum during immune attacks and afterwards (Wallerian degeneration leading to axonal loss) should be undertaken using stereology and neuroimaging techniques.

**Table 1:** Classification of immune-mediated cerebellar ataxias (IMCAs)

1. Autoimmunity targeting mainly the cerebellum^a^ or related structures^b^:
Cerebellar autoimmunity triggered by another disease or condition:
Gluten ataxia (gluten sensitivity)
Acute cerebellitis (infection)
Miller Fisher syndrome (infection)
Paraneoplastic cerebellar degenerations (neoplasm)
Cerebellar autoimmunity not triggered by another disease or condition:
Anti-GAD65 Ab-associated cerebellar ataxias ^c^
Steroid-responsive IMCAs with anti-thyroid antibodies
Primary autoimmune cerebellar ataxia (PACA)
Others
2. Autoimmunity that targets various parts of the CNS simultaneously:
Multiple sclerosis
Ataxia in the context of connective tissue diseases such as systemic lupus erythematosus

Modified from our consensus paper [2]

^a^ When cerebellar deficits are the sole or main symptoms, the cerebellum is presumed to be the main target of autoimmunity

^b^ Involvement of the proprioceptive spinocerebellar pathway is assumed in Miller Fisher syndrome

^c^ Excluding paraneoplastic patients

**Table 2:** Clinical features of the main types of immune-mediated cerebellar ataxias

	*Gluten ataxia*	*Paraneoplastic cerebellar degeneration*	*Anti-GAD65 Ab-associated cerebellar ataxia*
Gender	Women in 50–60% of patients		Mostly women (80–90%)
Mean age (years)	Mostly 40–50 (median 48)	26–85 (median 61)	Mostly 50–60 (mean 58)
Clinical course	Chronic/insidious	Subacute	Subacute or chronic/insidious
Cerebellar signs	Gait ataxia is predominant (100%), accompanied by upper limb (75%) and lower limb ataxia (90%), dysarthria (656%), and nystagmus (84%).	Pancerebellar cerebellar ataxias, which are sometimes preceded by nausea, vomiting and dizziness.	Gait ataxia is predominant (100%), accompanied by limb ataxia (71%), dysarthria (66%), and nystagmus (64%)
Other symptoms	Sensorimotor axonal neuropathy, gluten-sensitive enteropathy, gastrointestinal symptoms, focal myoclonus, palatal tremor, and opsoclonus	Malignancy, e.g. breast, uterus, ovaries, SCLC, Hodgkin’s disease, thymoma	Stiff-person syndrome, epilepsy, myasthenia gravis
Associated autoimmune diseases	Thyroiditis, T1DM, pernicious anemia	Not correlated	T1DM, thyroiditis, hemolytic anemia
Autoantibodies	Anti-gliadin Ab, TG2 Ab, TG6 Ab	Anti-Yo, Hu, Tr, CV2, Ri, Ma2, and VGCC(P/Qtype) Abs	Anti-GAD65 Ab, TPO, TG, ANA: 30/41 (73%)
Cerebellar atrophy on MRI	Normal or mild atrophy	Initially normal (during subacute phase)	Normal or mild atrophy

Epidemiological data are cited from our previous Consensus paper [2]

*Abbreviations*: *SCLC*, small cell lung carcinoma, *TG2 Ab and TG6 Ab* anti-transglutaminase 2 and 6 Abs

The aim of the present review is to discuss the diagnosis and therapeutic strategies of CAs based on the known pathophysiological and immunological mechanisms. First, we review recent progress in three immunological and physiological problems: “significance of autoantibodies”, “loss of immune tolerance”, and “switching from functional disorder to cell death”. Then, by utilizing this background, we attempt to present a comprehensive rationale for the diagnosis and early treatment of CAs. This review does not focus on MS due to complexity of the immune process and the heterogeneity of presentations [16].

## Significance of autoantibodies

### Autoantibodies associated with cerebellar ataxia: Cause or result?

IMCAs are characterized by their association with autoantibodies, but whether these autoantibodies are the cause or result of the IMCAs still remains a matter of debate. In a recent review, Lancaster and Dalmau classified three groups of neuronal autoantigens and discussed the immune mechanisms involved in the associated neurological diseases [17].

The first group includes nuclear and cytoplasmic proteins, such as Hu, Yo and M2. Due to the difficulty for Abs to access these intracellular antigens, T-cell mediated-immune responses are considered to play an important pathogenic mechanism for the neurological diseases in this group. These immune-mediated diseases show resistance to immunotherapies, especially in patients with anti-Yo and anti-Hu Abs [18]. Detection and quantification of autoantibodies can be used as a tool for the diagnosis (biomarker) and assessment of prognosis.

The second group of autoantigens includes cell surface proteins, especially synaptic proteins, such as voltage-gated potassium channels (VGKCs), glutamate receptors (AMPA, NMDA and metabotropic glutamate receptors), GABA_B_ receptor, and glycine receptor. Autoantibodies to these antigens presumably cause neurological symptoms. These diseases characteristically respond well to immunotherapy, which is an argument for a direct pathogenic role of Abs.

The third group of autoantigens includes intracellular synaptic antigens, such as GAD65 and amphiphysin. In addition to T-cell mediated pathogenic mechanisms (as in group 1), Abs are considered to be involved based on accessibility of the antigens to the Abs during synaptic vesicle fusion and reuptake.

### Characterization of actions of autoantibodies

In order to confirm that an autoantibody plays a pathogenic role in CA, it should fulfill two criteria: (1) it elicits effects that can lead to the development of ataxias or cell death, and (2) passive transfer of the antibody results in changes that mimic CA symptoms in vivo. Here we will review recent studies that analyzed the pathogenic effects of such autoantibodies and discuss how far each category of the autoantibodies satisfied the above two criteria.

#### Autoantibodies of the first group

Most types of PCDs exhibit intracellular antigens, suggesting that autoantibodies are unlikely to be involved in the pathogenesis of PCDs. Furthermore, passive transfer experiments using onconeuronal antibodies and immunization using protein or DNA failed to induce ataxic symptoms in animals [19–21]. High proportions of cdr2- or Hu-specific T cells are present in blood of patients with anti-Yo (cdr2) Ab or anti-Hu Ab, respectively [22–24], suggesting that PCDs are mediated by T-cell immune response towards an autoantigen recognized by onconeuronal Ab [5]. However, other investigators have challenged the above data. First, evidence now suggests that various autoantibodies can have access to intracellular antigens. Although previous studies ruled out possible internalization of antibodies [25], recent experiments have shown internalization of the antibodies into living cells, especially Purkinje cells (a masterpiece in the cerebellar cortical circuitry), under physiological conditions that are dependent on cell activities [26–31]. Second, pathogenic mechanisms of autoantibodies have been established recently. Cdr2, the target molecule of anti-Yo Ab, encodes a leucine zipper motif that interacts with another leucine zipper motif on c-Myc, a nuclear transcription factor [32]. Okano et al. [32] showed that anti-Yo Ab inhibits the interaction between Cdr2 and c-Myc, which results in excess c-Myc. The excess c-Myc is assumed to enter the nucleus and impair signals of cell cycling [32]. Similarly, the binding of anti-Yo antibodies to Cdr2 may prevent interaction with mortality factor-like proteins, MRGX and/or NFkb, and consequently alter the transcriptional activity and lead to cell apoptosis [33]. On the other hand, De Giorgio et al. [34] reported that anti-Hu Ab induce apoptosis when applied to cultures of neuroblastoma or myenteric cells. The above studies suggest that the intra-cellular location of antigens does not necessarily prevent pathogenic actions of autoantibodies directed against them.

In conclusion, internalization of autoantibodies and potential pathogenic actions by anti-Yo and Hu Abs challenge the classic notion that autoantibodies targeting intracellular antigens cannot be pathogenic. However, there is currently no convincing evidence that passive transfer of autoantibodies elicits PCDs. Thus, the significance of autoantibodies remains uncertain at this stage, especially from a clinical standpoint.

#### Autoantibodies of the second group

These types of autoantibodies are known to play a pathogenic role in limbic encephalitis [35]. Patients with limbic encephalitis develop epilepsy or complex clinical neuropsychiatric features, including memory and cognitive deficits, psychosis, seizures, movement disorders and coma [35].While paraneoplastic autoantibodies are the culprit in some patients, the cause in the majority of cases is undetermined or idiopathic [36]. Autoimmune encephalitis is associated with autoantibodies to the extracellular epitopes of receptors or proteins. The target molecules are involved in synaptic transmission and plasticity, and include voltage-gated potassium channel complex (LGI1, CASPR2) [37, 38], AMPA [39], NMDA [40] and GABA_B_ [41] receptors. The pathogenic effects are a direct down-stream effect of the recognition of these antigens by the respective autoantibodies. For example, autoantibodies directed towards AMPA and NMDA receptors decrease the numbers of these cell-surface receptors (internalization), potentially leading to behavioral deficit [42–44]. Autoantibodies to AMPA receptor also act as agonists and increase cell excitability [45]. The above studies conducted in the hippocampus have established the pathogenic roles of autoantibodies that target proteins involved in neuron excitability or synaptic transmission in the development of clinically overt neurological diseases.

Compared with autoimmune encephalitis of the limbic system, the second group of autoantibodies, such as anti-voltage gated calcium channel (VGCC) Ab, anti-metabotrophic glutamate receptors 1 (mGluR1), and anti-glutamate receptors delta2 (GluRδ2)-Abs, are less frequently involved in IMCAs. Anti-VGCC Ab is associated mainly with PCDs, especially small cell lung carcinoma [8]. Application of polyclonal Abs that target a major epitope in P/Q-type VGCC inhibits VGCC function of neurons and recombinant and causes ataxia in mice [46]. Previous studies described two patients with malignant lymphoma and one patient with prostate adenocarcinoma who were positive for anti-mGluR1 Ab [7, 47], but similar cases were not reported subsequently [7]. Thus, it is not clear at this stage whether anti-mGluR1 is a genuine onconeuronal Ab [2]. However, application of anti-mGluR1 impairs the induction of long-term depression (LTD), which causes ataxic behavior in mice [47]. On the other hand, the association of anti- GluRδ2 Ab with CAs was noted to be preceded by either infection or vaccination [8]. Injection of polyclonal Abs towards the putative ligand-binding site of GluRδ2 caused endocytosis of AMPA receptors and attenuated their synaptic transmission, resulting in the development of an ataxic phenotype in mice [48].

Taken together, the above studies have confirmed that the concept advocated by Lancaster and Dalmau can be applied also in IMCAs. It has been experimentally confirmed that antibody-mediated blockade of channels or receptors does not only impair signal formation in the cerebellar cortex (e.g., inputs or outputs to Purkinje cells), but also synaptic modulation or plasticity involved in motor control or learning, leading to the development of ataxic disorders in animals.

#### Autoantibodies of the third group

Accumulating evidence suggests a pathogenic role of anti-GAD65 Ab in the development of CAs [49–58]. It should be acknowledged here that the pathogenic role of anti-GAD65 Ab does not exclude at all the involvement of other Abs-mediated or cell-mediated immune mechanisms in the development of CAs.

Dinkel et al. [59] were the first group to report the pathogenic role of anti-GAD65 Ab in CAs. They found that anti-GAD65 Ab obtained from patients with stiff-person syndrome (SPS) inhibited GAD enzyme activities. Since then, many studies have confirmed the pathogenic role of anti-GAD65 Ab in CA based on in vitro and in vivo studies using CSF IgGs from CA patients and human monoclonal anti GAD65 Ab [49–58].

##### Dysfunction of synaptic transmission

Anti-GAD65 Ab binds to a shared GAD65 epitope and thus interferes with the association of GAD65 with cytosolic face of GABA-containing synaptic vesicles [53, 57]. Dissociation of GAD65 and GABA-containing vesicles results in impairment of GABA packaging into the vesicles and shuttling of vesicles to the release site on the synaptic cleft [57], which results in decreased GABA content in the vesicle and low release probability of the vesicle [57]. Previous studies confirmed the internalization of human monoclonal GAD65Ab b78 using cultured AF5 cells [57]. Moreover, b78 was internalized in PCs shortly after its injection into the ipsilateral interpositus nucleus [57].

##### Dysfunction of cerebellar circuits

**Fig. 1:**
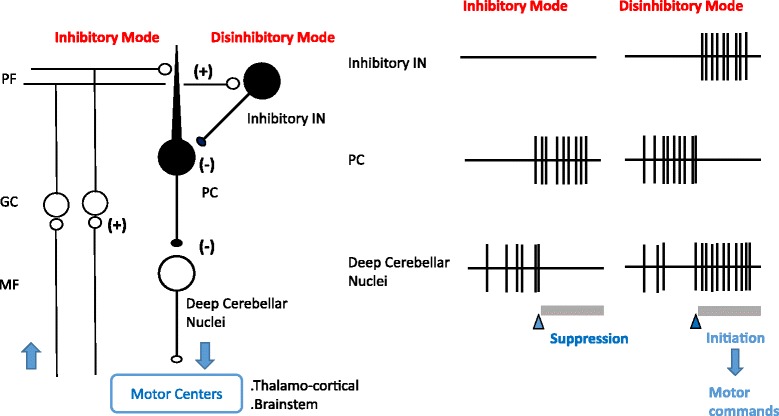
Signal formation by chained GABAergic interneurons in the cerebellar cortex. *Left panel:* Schematic diagram of the cerebellar cortex. Arrows: signal flows in the cerebellar cortex. White cells: excitatory neurons, black cells: inhibitory neurons. (+); excitatory synapses, (−); inhibitory synapses, MF; mossy fibers, GC; granule cells, PF; parallel fibers, Inhibitory IN; inhibitory interneurons, PC; Purkinje cells; Deep Cerebellar Nuclei; deep cerebellar nuclei neurons. *Right panel*: Schematic diagram of activities of inhibitory interneurons, Purkinje cells, and deep cerebellar nuclei neurons. MF inputs activate granule cells, which in turn elicit two modes, inhibitory mode and disinhibitory mode. *Inhibitory mode*; Purkinje cells inhibit activities of the output signals conveyed by cerebellar nucleus neurons, which suppresses adventitious movements. *Disinhibitory mod*e; Inhibition of Purkinje cells by inhibitory interneurons releases this inhibition on outputs, which facilitates the execution of aimed movements. Since this inhibitory/disinhibitory mode, which is formed by the chained GABAergic neurons, is an essential feature for cerebellar circuitry. A decrease in GABA release impairs signals formation in the cerebellum, causing cerebellar ataxia through abnormal motoneuronal commands

In cerebellar circuits, Purkinje cells inhibit the activities of output signals conveyed by the cerebellar nucleus neurons (inhibitory mode), which results in the suppression of adventitious movements. Inhibition of Purkinje cells by inhibitory interneurons releases this inhibition on outputs (disinhibitory mode), which facilitates the execution of aimed movements. Since this inhibitory/disinhibitory mode, which is formed by chained GABAergic neurons, is a major mechanism of cerebellar control of movement (Fig. 1) [60], the decrease in GABA release [49–52] can interfere with the genesis of motor commands in the circuits of the cerebellar cortex.

##### Disorganization of cerebellar motor control

Impairment of command formation ends up in disorganized cerebellar control in various motor control centers. Consistently, intracerebellar administration of anti-GAD65 Ab elicits impairment of cerebellar modulation of motor cortex activities, conditioned eyelid responses, and gait [54–57].

In summary, the above studies clearly show that anti-GAD65 Ab target GAD65 molecules involved in GABA release, leading to dysfunction of cerebellar circuits and deficits in cerebellar motor control. In vitro and in vivo studies have demonstrated the accessibility of anti-GAD65 Ab to GAD65 at the cytosolic face of GABAergic synaptic vesicles. It is hypothesized that anti-GAD65 Ab interact with GAD65 during exocytosis, when the antigen is temporarily exposed, and thus accessible to anti-GAD65 Ab [61, 62]. Other studies reported the association of anti-GAD65 Ab with stiff-person like syndromes in dogs and horses [63]. This association supports the hypothesis that anti-GAD65 Ab are a genuine trigger of neurological symptoms.

#### Cases with non-identifiable antigens

GA is associated with anti- transglutaminase 2 (TG2) Ab that targets TG2 present in most tissues, and anti-transglutaminase 6 (TG6) Ab against TG6 present in the CNS [64]. Tissue transglutaminase is a calcium-dependent enzyme which crosslinks proteins, and is located in intra- and extra-cellular spaces. Several in vitro and in vivo studies have confirmed the pathogenic role of anti-TG Abs. A subpopulation of anti-TG2 antibodies in celiac patients recognizes neurons and cross-reacts with TG6. Anti-TG2 Abs obtained from patients with celiac disease induced apoptosis of cultured neuronal cells [65]. Notably, intraventricular injection of anti-TG2 or anti-TG2/6 cross-reactive Abs induced ataxia in mice [66]. However, it is not clear whether the Abs block tissue transglutaminase in the intracellular or extracellular space.

TG2 activity is up-regulated through the binding of calcium. Anti-TG2 Abs cloned from patients with celiac disease bind preferentially to the calcium-activated enzyme conformation [67], suggesting that deficits of TG2 in its enzymatically active conformation may be the mechanism underlying the pathogenic actions of anti-TG2/6 Abs. This hypothesis is supported by the presence of aberrant cerebral TG activity and protein aggregates in the affected brain regions of patients with neurodegenerative diseases [68].

#### Conclusion

Recent studies have shown that autoantibodies targeting channels and receptors on the cell surface, as well as GAD65 on synaptic vesicles can induce pathogenic changes both in vitro and in vivo preparations. These pathomechanisms are mediated by autoantibodies-induced impairments in neuron excitability or synaptic transmission/modulation.

Regarding onconeuronal autoantibodies in PCD, given that the antibodies can be internalized, their pathogenic importance cannot be ignored as they target intracellular antigens and intra-cellular events. Studies examining the effects of passive transfer are necessary in order to confirm the pathogenic role of each autoantibody. For onconeuronal autoantibodies, the results of passive transfer experiments have been negative so far. However, reproduction of CAs in vivo might be experimentally difficult, except in cases where the autoantibodies target molecules involved in neuronal excitability or synaptic dysfunction. Further technical progress in vivo will be necessary to confirm the pathogenic roles of autoantibodies that target intracellular antigens.

Autoantibodies against tissue transglutaminases appear to elicit CAs, although the location of the target molecules (i.e., intracellular or extracellular), is not clear.

Namely, while the location of an antigen can allude to the associated pathomechanism, the pathogenic role of the autoantibodies can be confirmed only by clear actions in both in vitro and in vivo experiments. The correlation between cell-mediated and autoantibodies-mediated pathogenesis remains to be clarified.

### Epitope-specific actions of autoantibodies

The epitope specificities of autoantibodies should be taken into consideration when estimating their clinical significance. Initially, the pathogenicity of anti-GAD65 Ab was questioned [69], because the antibodies are associated with different disease phenotypes, including CAs, SPS and type 1 diabetes mellitus (T1DM) [70, 71]. A series of experiments have demonstrated the pathogenic impact of anti-GAD65 Ab with distinct epitope specificities, revealing that the epitope specificity of anti-GAD65 Ab determines the phenotype of neurological symptoms [54–58].

#### Epitope and actions of anti-GAD65 Ab obtained from patients

Anti-GAD65 Ab titers in patients with SPS and CAs are significantly (500-fold) higher than in patients with T1DM. Moreover anti-GAD65 Ab recognizes both conformational and linear epitopes in SPS and CA, while anti-GAD65 Ab epitopes in T1DM are strictly conformational [72–74]. Anti-GAD65 Ab elicits pathogenic changes in in vivo and in vitro CAs studies, but not in T1DM [49, 54, 57].

#### Epitope and actions of human monoclonal GAD65 Ab

Studies using human monoclonal Ab have provided more detailed epitope-action relations [55–57]. Human monoclonal GAD65 Ab b96.11 binds to an epitope that is recognized by anti-GAD65 Ab in T1DM patients [55, 75], whereas human monoclonal anti-GAD65Ab b78 binds to an epitope that is recognized by anti-GAD65 Ab in SPS and CAs [55, 57]. Importantly, both monoclonal anti-GAD65 Abs recognize epitopes spanning the middle and the C-terminal region of GAD65; however, the respective conformational epitopes are distinctly different [76]. Based on the differences in the epitope specificities, the actions of anti-GAD65 Ab in CAs are closely mimicked by b78, rather than by b96.11 [55–57].

#### Epitope and neurological phenotype

Differences in neurological phenotypes (i.e., CAs and SPS), can be explained by differences in the epitope-dependent actions of anti-GAD65 Ab. Anti-GAD65 Ab in CAs interferes with GABA release [55, 57], which results in disorganization of –mainly- the phasic discharges in the cerebro-cerebellar loop, and ultimately leads to the development of CAs. On the other hand, anti-GAD65 Ab impairs GABA synthesis in SPS [55, 59, 72], which results in interference with the tonic suppression of cerebellar nuclei in the spino-cerebellar loop, leading to hyperexcitability of spinal motoneurons. These assumptions were confirmed by injection of CSF IgGs obtained from CAs and SPS patients into experimental animals [55].

In conclusion, epitope specific polyclonal autoantibodies are associated with different pathological conditions [56]. The pathologic phenotype will be determined based on the proportion of epitope-specific pathogenic autoantibodies [56].

### Clinical significance of autoantibodies from diagnostic and therapeutic points of view

Based on basic research findings, we will discuss here the clinical significance of autoantibodies in diagnosis and treatment.

#### Issues in diagnosis

Regardless of the roles of autoantibodies in the pathogenesis, autoantibodies are considered as markers of specific diseases. However, it should be acknowledged that diagnosis based purely on the detection of autoantibodies is sometimes misleading.

The association of onconeuronal antibodies, such as anti-Yo, anti-CV2, anti-Ri, and anti-MA2 Abs, in the diagnosis of PCD is well established. According to the guidelines of “Recommended Diagnostic Criteria For Paraneoplastic Neurological Syndromes 2004”, the diagnosis of classical paraneoplastic neurological syndromes depends on the presence of a neoplasm that develops within 5 years of the diagnosis of CAs, or the presence of onconeuronal antibodies [4]. The presence of onconeuronal Abs sometimes suggests a specific type of neoplasm [4–9] (Table 3). However, the diagnosis should not depend solely on the presence of such Ab, since some patients show no association with onconeuronal Abs (seronegative PCDs) [5, 6].

**Table 3:** Representative autoantibodies to cerebellar antigens in paraneoplastic cerebellar degenerations

Autoantibodies	Frequency in PCDs	Localization of antigens
Anti-Yo	53%, breast, uterus, ovaries	Mainly Purkinje cells and few other neurons in the molecular layer
Anti-Hu	15%, SCLC	All neuronal nuclei and cytoplasm
Anti-Tr	5%, Hodgkin’s disease	Purkinje cells cytoplasm and dendrites,
Anti-CV2	4%, SCLC, thymoma	Oligodendrocytes
Anti-Ri	2%, Breast	All neuronal nuclei
Anti-Ma2	2%, Testes, Lung	Nucleoli
Anti-VGCC(P/Q type)	2%, SCLC	Purkinje cells, cytoplasm, dendrites and dot-staining of the molecular layer

Frequency among PCDs was evaluated based on our consensus paper [2]

Localization was based on a review by Jarius and Wildemann [7–9]

Modified from Mitoma et al. (2016) [2]

*Abbreviations*: *SCLC* small cell lung carcinoma

On the other hand, in GA and anti-GAD65 Ab-associated CA, low antibody specificity or low antibody titers may pose a problem in the differential diagnosis. In GA, anti-gliadin Ab is currently used for the diagnosis. However, as shown by Hadjivassiliou et al. [77], anti-gliadin Ab have low disease-specificity and are detected also in 14% of familial degenerative CAs, 15% of MSA-C patients and 12% of healthy subjects. A more evident example is found in anti-GAD65 Ab-associated CA. Some CA patients have a low-titer (<100 U/ml) of anti-GAD65 Ab [78]. These patients respond well to immunotherapies, exhibiting a clinical improvement. However, there is general agreement that these patients should not be categorized as anti-GAD65 Ab-associated CA [14, 78], since anti-GAD65 Ab is not necessarily produced intrathecally and its titer does not correlate with clinical improvement [14, 78]. The findings that CSF obtained from these patients had no effect on cerebellar transmission in slices [unpublished data] suggest that there are differences in epitope specificities between high-titer and low-titer anti-GAD65 Abs. This problem of false positivity might be due, at least in part, to differences in autoantibody epitope specificities.

The diagnosis of each subtype of IMCAs should be based firstly on the overall clinical profile (Table 2). When clinicians encounter a patient with subacute pancerebellar CAs, PCDs should be considered in the differential diagnosis. Although 70% of the patients showed neurological symptoms as the initial symptoms in paraneoplastic syndromes [5], CAs are sometimes preceded by prodromal symptoms, such as nausea, vomiting, or dizziness [5] and subsequent examination of the CSF is needed to determine CNS inflammation, including moderate lymphocyte pleocytosis, high protein levels, high IgG index, and CSF-specific oligoclonal bands [5]. If PCD is clinically suspected, it is recommended that a whole body tumor search should be performed even when no onconeuronal antibody is detected [79]. On the other hand, GA and anti-GAD65 Ab-associated CA should be listed in the differential diagnosis, when patients, especially women aged 40–60s, show CAs with chronic and insidious clinical course [2]. The main symptom is gait ataxia, which is usually prominent compared with the degree of cerebellar atrophy [2]. GA is often associated with other autoimmune diseases, such as thyroiditis, T1DM, and pernicious anemia [10], whereas anti-GAD65 Ab-associated CA is frequently associated with T1DM [11]. Thus, comprehensive assessment of neurological symptoms and autoantibodies is necessary for a definite diagnosis.

#### Issues in therapies

In patients with pathogenic Abs, the first aim of the treatment is to reduce the titers of these Abs. Indeed, in anti-GAD65 Ab-associated CA, clinical improvement correlates with reduction in anti-GAD65 Ab titers [14]. For example, immunotherapy resulted in marked reduction of anti-GAD65 Ab titers in 10 of the 14 patients who showed response within a short-term observation period [14]. Furthermore, a combination of immunotherapeutic agents are recommended until a reduction in anti-GAD65 Ab titer or CAs symptoms are observed [14] (Table 4). Notably, the therapeutic benefits of plasma exchange and rituximab has been confirmed [14]. The therapeutic benefits were observed in 4 of 6 patients, who received plasma exchange and 3 of 5 patients, who were treated with rituximab [14]. These results suggest promising benefits from therapies designed to absorb and inhibit the synthesis of Abs. Further developments in this area are expected in the next few years.

**Table 4:** First-line immunotherapy for the main subtypes of immune-mediated cerebellar ataxia

Gluten ataxia
Induction and maintenance therapies: strict gluten-free diet
Immunosuppressants or IVIg for patients who show no improvement or are negative for gluten-related antibodies
Paraneoplastic cerebellar degeneration
Early removal of neoplasm is the first objective of treatment, followed by induction therapy (mPSL, IVIg, immunosuppressants, or/and plasma exchange). Discussion according to associated Abs. Long-term oral PSL, IVIg, immunosuppressants for maintenance therapy
Anti-GAD65 Ab-associated cerebellar ataxia
Induction therapy: mPSL, IVIg, immunosuppressants, plasma exchange, or/and rituximab
Maintenance therapy: continuous oral PSL, IVIg, immunosuppressants, or/and rituximab

Modified from Mitoma and Manto (2016) [14]

*Abbreviations*: *Abs* antibodies, *mPSL* intravenous methylprednisolone, *oral PSL* oral prednisolone, *IVIg* intravenous immunoglobulins

## Loss of immune tolerance

### Possible mechanisms that trigger autoimmunity in GA, PCDs, and anti-GAD65 Ab-associated CA

Lymphocytes that react with self-antigens are eliminated by negative-selection via apoptosis facilitated by clonal deletion [80]. Autoimmunity is also suppressed by regulatory T cells [81]. These central and peripheral immune tolerance mechanisms can avoid an immune attack of the CNS. On the other hand, several mechanisms are involved in the loss of immune tolerance in IMCAs.

#### Loss of tolerance in GA

Autoimmunity is often triggered by exposure to chemicals or dietary components. Gluten sensitivity is an example in which a diet component becomes a potential environmental risk, leading to activation of autoimmunity. Gluten sensitivity has been examined in detail in celiac disease [82]. Digested gluten peptides are cross-linked and deaminated by transglutaminase 2 (TG2), leading to the creation of an immunostimulatory epitope for HLA-DQ2 or -DQ8 on antigen-presenting cells. These epitopes are presented to CD4^+^ T cells, from which cytokines are released to facilitate the production of antibodies against gliadin and TG2. Similar mechanisms are assumed to operate in GA.

#### Loss of tolerance in PCDs

Autoantibodies against intracellular antigens are characteristically produced in association with malignancies. The following mechanisms are considered to be involved in autoimmunity [83]: 1) Downregulation of regulatory T cells; 2) persistent autoimmune response to the malignancy. The released cytokines cause chronic inflammation with vascular hyperpermeability. This microenvironment facilitates access of immune cells; 3) structural modification of some intracellular proteins in cancer cells which can lead to stimulation of the immune system. Somatic mutations change the structural motif of intracellular antigens and intracellular proteins are prone to post-translational modifications; 4) death of cancer cells which results in the release of intracellular antigens and subsequent exposure to the immune system.

Autoimmunity and production of autoantibodies against intracellular antigens progress to a chronic state with the growth of the malignant tumor, and probably explains the observed resistance to immunotherapies, as discussed in the next chapter.

#### Loss of tolerance in anti-GAD65 Ab-associated CA

Molecular mimicry is a typical example of loss of peripheral immune tolerance [84]. The immune response to microbial antigens can show cross-reaction towards similar antigens in nervous systems. The homology of Coxsackie B4 virus and GAD65 has been suggested to underlie the development of T1DM [85]. However, the clinical course of T1DM differs from that of anti-GAD65 Ab-associated CA. The former shows acute or subacute clinical course, whereas it is more chronic in the latter. A few studies also suggested a positive history of infection before the development of CAs. Further studies are needed to determine the importance of molecular mimicry in the development of neurological symptoms.

In conclusion, various mechanisms are thought to trigger autoimmunity in IMCAs, thus reflecting the divergent clinical features of these forms of ataxia.

### Therapeutic principle: removal of triggering factors

The autoimmune mechanisms discussed in the above chapter suggest that when autoimmunity is triggered by certain conditions or diseases, removal of the triggering factor should be the first line of treatment.

In GA, the first line of treatment is a gluten-free diet [14, 86]. Most patients with GA show good response after one year of gluten-free diet [14, 86]. Although there are reports that intravenous immunoglobulins (IVIg) are effective in patients resistance to gluten-free diet [87, 88], the main reason for lack of response is poor adherence to the diet or hypersensitivity to gluten in which a small amount of gluten is still contained in the commercially available gluten-free food [89, 90]. Such patients have high titers of anti-gliadin Ab [89, 90]. Thus, it is recommended that gluten-free diet should be monitored by repetitive measurement of anti-gliadin Ab titer. In patients with high titers, strict adherence to gluten-free diet or wheat-free diet should be considered [89, 90] so as to restrict antigens that can trigger autoimmune reactions.

In PCDs, the first line of therapy is surgical excision of the tumor as soon as possible [4–6]. Depending on the type of malignancy, surgery can be combined with radiotherapy and/or chemotherapy [4–6], and is later followed by the use of various kinds of immunotherapies (corticosteroids, IVIg, plasmapheresis, immunosuppressants, and rituximab, alone or in combination). However, in contrast to GA, which carries good prognosis under strict gluten-free diet, the prognosis of PCDs is relatively poor [4–9]. For example, one long-term study reported that 23% of patients with PCDs died before the final follow-up, with a mean survival time from the onset of CAs of 42 months [91]. Another long-term study reported that 88% of the patients with PCDs died during the follow-up period due to metastasis (56% of causes of death) or neurological deficits (19%), with a mean survival time of 10.2 months [92]. Furthermore, most of the surviving patients show no improvement in CAs even after combination immunotherapies [5, 93, 94]. These results suggest that metastasis is problematic, as it can result in multi-organ failure and persistent simulation of autoimmunity. In addition, since various mechanisms can trigger neoplasm-induced autoimmunity, it is may be difficult to control immune reactions.

Although the prognosis after immunotherapy is different between GA and PCDs, the results of treatment of both conditions suggests the importance of removal of the antigens. It is possible that therapies designed to avoid the loss of tolerance could have a better chance in improvement of prognosis.

## Switching from functional disorder to cell death

### Existence of restorable and non-restorable stage

Patients with PCDs usually exhibit no evident cerebellar atrophy during the subacute clinical course [4–9]. On the other hand, patients with GA and anti-GAD65 Ab-associated CA show progressive atrophy of the cerebellum during the clinical course [10–12]. These distinct clinical courses suggest that autoimmune attacks firstly induce functional disorders of the cerebellar motor and cognitive controls, which are subsequently followed by cell loss.

The degree of the atrophy affects the response to immunotherapy. GA and anti-GAD65 Ab-associated CA are diseases that respond to immunotherapy, i.e., immunotherapy can potentially prevent the progression of immune-mediated response [10–12]. Interestingly, two scenarios are observed during the clinical course after the cessation of autoimmune progression. Administration of immunotherapies during the lack of or the presence of only mild cerebellar atrophy results in improvement of CA either partially or completely [10–12]. On the other hand, delayed administration of immunotherapies until the development of marked cerebellar atrophy results in persistence of CAs, though no progress occurs in the disease process [10–12, 14].

Based on these features, we proposed recently the novel idea of “restorable stage/non-restorable stage” [95]. We hypothesize that self-recovery capacity is preserved within the restorable stage [95]. The cerebellum is endowed with various types of synaptic plasticities [96], and various modalities of sensory information converge on a single microcomplex, the cerebellar functional unit [97]. These dynamic features constitute the “cerebellar reserve”. The “cerebellar reserve” is thought to be preserved during the functional disorder and mild cell loss stages. As for other disorders affecting the CNS, time is a key-factor and all efforts should be made to identify/treat the IMCA as quickly as possible.

### Mechanisms underlying switching from functional disorder to cell death

The mechanisms of GABAergic synaptic dysfunction could provide a general mechanism of switching from a functional disorder to cell loss. As discussed above, anti-GAD65 Ab reduce the level of GABA in cerebellar GABAergic neurons. Since the released GABA spills over into neighboring GABA_B_ receptors on glutamatergic synapses, resulting in presynaptic inhibition of glutamate release, a decrease in GABA release elicits an increase in glutamate release [52]. Consequently, the marked imbalance between glutamate and GABA (increase in glutamate release and decrease in GABA release), will result in the development of the following cascade of reactions:

#### Activation of NMDA receptors and Ca^2+^ influx

Excessive release of glutamate activates a large number of post-synaptic NMDA receptors [98]. Especially through the activation of extrasynaptic NMDA receptors, the increased net Ca^2+^ influx stimulates calpain I and nNOS, which elicit DNA and mitochondrial damage by the formation of ONOO^−^ (a free radical) [98].

#### Involvement of microglia and neuroinflammation

Glutamate stimulates microglia, which in turn release glutamate [99] and TNF-α, an inflammatory factor [100]. Glutamate might be released via a non-vesicular fashion through the xc(−) system on astrocytes, a cystine/glutamate antiporter exchanging extracellular cystine for the release of intra-cellular glutamate. It should be noted that the uptake of cystine is essential for the intra-cellular production of the antioxidant glutathione (GSH), a scavenger of reactive oxygen species (ROS) [101]. A deficit in intra-cellular cystine may thus lead to oxidative stress. The antiporter (which is expressed in astrocytes, microglia, ependymal cells, choroid plexus and leptomeninges) constitutes a non-vesicular route of glutamate release which is implicated in neuronal signaling and may contribute to the cascade of excitotoxicity by an overactivation of ionotropic glutamate receptors. Increased extra-cellular glutamate levels compromise the activity of the system xc(−), resulting in GSH depletion and cell death.

On the other hand, TNF-α inhibits glutamate transport on astrocytes, one main mechanism for physiological clearance of extracellular glutamate [100]. Through these pathways glutamate-induced activation of microglia may lead to accumulation of glutamate in a positive feed-back fashion. In addition to the effects of glutamate accumulation, TNF-α also triggers excitotoxicity by increasing the expression of Ca^2+^ permeable AMPA receptors and NMDA receptors [102, 103].

In summary, the anti-GAD65 Ab-induced decrease in GABA release causes high glutamate levels, a process accelerated through a positive feed-back loop, leading to excitotoxicity with resultant DNA damage, oxidative stress, and mitochondria damage. These positive-feedback spiral reactions might underlie the switching from functional disorders to cell death. Under such conditions, once the stage is beyond the threshold, accelerated cell loss is anticipated. Consistently, pathological examination in the advanced stage often shows marked cell loss in the cerebellar cortex [104, 105].

Both common and different features are encountered between anti-GAD65 Ab in IMCAs and autoantibodies directed against glutamate receptors in limbic encephalitis [43, 44] (Table 5). The common features are that the autoantibodies impair synaptic transmission. On the other hand, the most outstanding difference between these two synaptic dysfunction diseases are that anti-GAD65 Ab elicits a marked imbalance between glutamate and GABA, with relative predominance of glutamate over GABA, whereas anti-glutamate receptors Abs elicit impairment of glutamate transmission only without causing imbalance in the two neurotransmitters. The former change would lead to cell death where intensive immunotherapies are not effective, while the latter change limits the damage to a functional disorder in which self-recovery is possible. These differences could be due to differences in the target site, presynaptic site (release mechanisms) or postsynaptic site (receptors).

**Table 5:** Comparison between anti-GAD65 antibody and anti-glutamate receptors antibodies

	Anti-GAD65 antibody	Anti-NMDA and anti-AMPA receptors antibodies
Common features	Autoantibodies-induced synaptic dysfunction	
Action site	Presynaptic site	Postsynaptic site
Actions	Decrease in GABA release due to impaired release mechanisms	Decrease in receptors due to internalization
Functional impairment in synaptic transmission	Impaired inhibitory synaptic transmission	Impaired excitatory synaptic transmission
Imbalance between glutamate and GABA	Predominance of glutamate over GABA	Glutamatergic dysfunction: Impairment of both the NMDA- and the AMPA-mediated synaptic regulation of glutamate. No effect on the glial transport of glutamate^a^.
Excitotoxicity	Prompt excitotoxicity leading to cell death	Probable excitotoxicity
Immunotherapy	Not effective cases with atrophy and poor prognosis	Effective and good prognosis at an early stage

^a^Evidence in CA1 area of Ammon’s horn and in premotor cortex in rats

### Therapeutic principles: early intervention with immunotherapy

Further studies are needed to determine whether switching from a functional disorder to cell loss underlie all subtypes of IMCAs. However, since similar switching is also proposed in degenerative CAs [106], the switching could probably be generalized to IMCAs. The importance of early intervention using appropriate immunotherapy has been clearly confirmed in systemic reviews of the therapeutic effects in case reports [14]. Based on the abovementioned mechanisms responsible for the progression from functional disorders to cell loss in anti-GAD65 Ab-associated CA, we propose the following therapeutic strategies.

The first aim of immunotherapy is to halt the progression of autoimmune CA within the period of preservation of “cerebellar reserve” [95]. For this purpose, strict use of gluten-free diet and removal of antigens form the backbone therapy in GA [107–109]. In PCDs, removal of the tumor as soon as possible should be attempted, followed by intensive immunotherapy with corticosteroids, IVIg, plasmapheresis, immunosuppressants, or rituximab, either alone or in various combinations [4–6]. Voltz [110] proposed a regime of immunotherapy consisting of one course of intravenous methyl prednisolone, followed by 1 or 2 weeks of IVIg in case of lack of improvement, and plasmapheresis or cyclophosphamide in case of no efficacy in the following 1 or 2 weeks. Dalmau and Rosenfeld [5] showed that the combination of IVIg or plasmapheresis with cyclophosphamide was effective in a subgroup of patients. On the other hand, in anti-GAD65 Ab-associated CA, in which no clear triggering antigens are identified, intensive combination immunotherapies are recommended until retardation of CA progression [14]. The combined immunotherapies include corticosteroids, IVIg, plasmapheresis, immunosuppressants, or rituximab, either alone or in various combinations. [14].

After a successful arrest of autoimmune-mediated progression, the second aim of therapy should be to restore CAs by potentiating “cerebellar reserve”, using motor rehabilitation or noninvasive cerebellar stimulation [14]. Importantly, this therapeutic strategy depends on early diagnosis. Thus, early diagnosis and therapy is critical in IMCAs. Since measurement of specific autoantibodies is diagnostically helpful in many cases, what is most important is the suspicion of IMCAs.

## Conclusion

A new category of IMCAs was established during the last three decades. IMCAs are induced through various etiologies, including PCDs, GA, and anti-GAD65 Ab-associated CA. Autoantibodies are usually associated with each subtype. Anti-GAD65 Ab diminishes GABA release, thus resulting in CAs, as confirmed in both in vitro and in vivo preparations. Accumulating evidence indicate the involvement of anti-TG2Ab in the pathogenesis of GA. On the other hand, the significance of onconeuronal autoantibodies in PCDs is still uncertain, but they represent an important biomarker. It has been assumed that onconeuronal autoantibodies are not pathogenic, since they target intracellular antigens. However, the internalization of Ab and the pathogenic effect in vitro now challenges this theoretical notion. Since there is no substantial evidence of pathogenicity from in vivo studies, no consensus has been reached yet. Thus, there is a need to explore the pathogenic actions using both in in vitro and in vivo preparations. The involvement of cell-mediated immune mechanisms in various types of IMCAs should not be ruled out. Both effects are non-exclusive.

Several studies on the actions of anti-GAD65 Ab have suggested that autoantibodies elicit pathogenic actions according to epitope specificity, and that the clinical course can be divided into two stages functional disorder and cell death. These concepts provide the rationales for diagnosis and early therapeutic strategies. The diagnosis should not depend on Ab testing only, but should rather include comprehensive assessment of clinical profiles. With regard to therapy, early intervention will be necessary during the period of preservation of cerebellar reserve, defined as capacity to restore lost functions. It is recommended that 1) patients with conditions or diseases known to trigger autoimmunity should be treated first by removal of the cause, and (2) after the removal, a combination of various immunotherapies will be selected dependent on each cancer subtype.

One of the open questions is the problem: "how does autoimmunity elicit a specific phenotype of neurological symptoms?". For example, "why does the appearance of anti-GAD65 Ab mainly lead to cerebellar dysfunction and CAs or SPS? " Although the epitope specificity of Abs might be one mechanism, other yet unknown mechanisms could be involved in the development of a particular phenotype. It is expected that vulnerability of the cerebellar circuitry will be explained by more detailed cellular, molecular or even anatomical mechanisms. Such understanding could help select new diagnostic methods and therapeutic strategies.

## Abbreviations

Ab: Antibody
CA: Cerebellar ataxia
GA: Gluten ataxia
GAD: Glutamic acid decarboxylase
IMCAs: Immune-mediated cerebellar ataxias
IVIg: Intravenous immuneglobulins
mPSL: Intravenous methylprednisolone
MS: Multiple sclerosis
oral PSL: Oral prednisolone
PCDs: Paraneoplastic cerebellar degenerations
PCs: Purkinje cells
SCLC: Small cell lung carcinoma
SPS: Stiff-person syndrome
T1DM: type 1 diabetes mellitus
TG: Ttransglutaminase
VGKC: Voltage-gated potassium channels
